# Epigenetic Regulation of *WNT3A* Enhancer during Regeneration of Injured Cortical Neurons

**DOI:** 10.3390/ijms21051891

**Published:** 2020-03-10

**Authors:** Chu-Yuan Chang, Jui-Hung Hung, Liang-Wei Huang, Joye Li, Ka Shing Fung, Cheng-Fu Kao, Linyi Chen

**Affiliations:** 1 Institute of Molecular Medicine, National Tsing Hua University, Hsinchu 30013, Taiwan; chyju13180@hotmail.com (C.-Y.C.); kashing94@gmail.com (K.S.F.); 2 Department of Computer Science, National Chiao Tung University, Hsinchu 30010, Taiwan; juihunghung@gmail.com (J.-H.H.); joyeli@outlook.com (J.L.); 3 Department of Life Science, National Tsing Hua University, Hsinchu 30013, Taiwan; h211143@nehs.hc.edu.tw; 4 Institute of Cellular and Organismic Biology, Academia Sinica, Taipei 11574, Taiwan; ckao@gate.sinica.edu.tw; 5 Department of Medical Science, National Tsing Hua University, Hsinchu 30013, Taiwan

**Keywords:** enhancer regulation, histone modification, neuronal regeneration, epigenome, transcriptional regulation, WNT3A

## Abstract

Traumatic brain injury is known to reprogram the epigenome. Chromatin immunoprecipitation-sequencing of histone H3 lysine 27 acetylation (H3K27ac) and tri-methylation of histone H3 at lysine 4 (H3K4me3) marks was performed to address the transcriptional regulation of candidate regeneration-associated genes. In this study, we identify a novel enhancer region for induced *WNT3A* transcription during regeneration of injured cortical neurons. We further demonstrated an increased mono-methylation of histone H3 at lysine 4 (H3K4me1) modification at this enhancer concomitant with a topological interaction between sub-regions of this enhancer and with promoter of *WNT3A* gene. Together, this study reports a novel mechanism for *WNT3A* gene transcription and reveals a potential therapeutic intervention for neuronal regeneration.

## 1. Introduction

There are approximately 70 million individuals suffering from traumatic brain injury (TBI) each year, which has been a major cause of morbidity and lifelong disability worldwide [1]. TBI is caused by external mechanical force, such as a blow to the brain, or by other mechanisms of displacement of the brain tissue that leads to focal (e.g., penetrating injuries) or diffuse brain damage (e.g., blast exposure) [2]. Following primary injury, secondary injury can evolve and sustain complexed pathophysiological cascades. Secondary injury-initiated cascades include hypoxia, ischemia, increased neurotransmitter excitotoxicity (e.g., increased release of glutamate), diffuse neuronal depolarization and an excessive cellular influx of calcium, leading to cellular/molecular/metabolic derangements and ultimate cellular dysfunction or cell death [3]. In this regard, long-lasting and progressive nature of pathophysiology after TBI warrants secondary injury cascades a potential target for therapeutic intervention. Unfortunately, due to the heterogeneity of pathophysiology, severity, outcomes upon brain injury and the lack of knowledge about the underlying molecular mechanism, there is currently no therapy for TBI. Promising outcomes in preclinical studies did not lead to clinical efficacy. There are neither efficacy-proven neuroprotective nor neuroregenerative agents available to treat TBI in clinical trials [4]. Poor regeneration of the injured central nervous system in part due to inhibitory gliosis and an aging-related decline of intrinsic regenerative capacity [5,6,7]. However, inhibiting the non-permissive environment alone has not been a successful strategy to promote regeneration of injured brain neurons [8,9,10]. TBI was reported to reprogram epigenome [11,12]. Epigenomic profiling of mice hippocampus after TBI exposure revealed injury-induced inter-correlated transcriptomic and methylomic perturbations [12]. Significant epigenetic changes identified in the brain after TBI in animals and humans include DNA methylation, histone methylation/acetylation and microRNAs expression. Cognitive or functional recovery was assayed following administration of epigenetic modifying drugs in post-TBI animal models, demonstrating variable behavioral outcomes [11]. As more attention has been drawn on the epigenetic mechanism upon TBI, TBI-relevant epigenetic changes are found implicated in shaping neuronal functions and plasticity [13]. It is believed that better understating of the pathophysiology following brain injury in an epigenetic scale would gain insight into therapeutic development for TBI. To this point, we performed chromatin immunoprecipitation-sequencing (ChIP-seq) of histone marks, tri-methylation of histone H3 at lysine 4 (H3K4me3) and histone H3 lysine 27 acetylation (H3K27ac), to investigate transcriptional regulation of candidate regeneration-associated genes (RAGs). Our laboratory identified *WNT3A* gene as a promising RAG of which expression was up-regulated during regeneration of injured cortical neurons [14]. Nonetheless, the transcriptional regulation of *WNT3A* expression remains to be determined. This study aims to investigate the epigenetic regulation of induced *WNT3A* transcription via promoter and novel enhancer. Depending on the types of modifications (e.g., mono-/di-/tri-methylation, acetylation) on target residues, histone modifications may be active or suppressive to transcription [15]. For example, di-methylation of histone H3 at lysine 9 (H3K9me2) and tri-methylation of histone H3 at lysine 27 (H3K27me3) are classified as repressive histone modifiers. Up-regulation of known RAGs, such as *BDNF* and *Galanin*, after sciatic nerve axotomy coincides with decreased H3K9me2 at the promoters [16,17]. On the contrary, H3K4me3 is typically associated with active promoter, which drives RAGs expression in the CNS [18,19]. H3K4me3 was reported to be enriched in neurodevelopmental genes in progenitor cells within primate subventricular zone (SVZ), a brain region where neurogenesis occurs [20,21]. Genomic enhancers are typically marked by mono-methylation of histone H3 at lysine 4 (H3K4me1) (prime) and additional H3K27ac upon activation. Moreover, active enhancers are frequently bound by acetyltransferase P300. Poised enhancers represent bivalent chromatin properties by carrying both H3K4me1 and H3K27me3 modifications that are associated with active and repressed chromatin states, respectively [22]. The abundance of specific chromatin signatures, such as enriched H3K27ac or a high ratio of histone H3K4me1 to H3K4me3, suggests that enhancers far outnumber protein-coding genes in the human genome, reflecting their critical role in transcriptional control, and is widely regarded as the hallmark for enhancer prediction [23,24].

## 2. Results

### 2.1. Identification of Putative Enhancer for WNT3A

**Figure 1:**
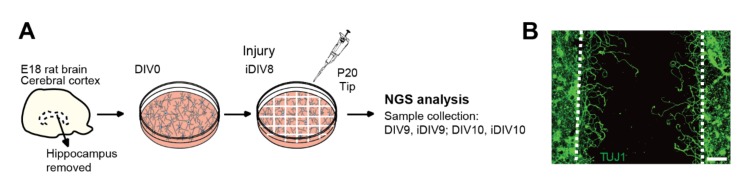
In vitro traumatic brain injury (TBI) model used in this study. (**A**) Schematic overview of the chromatin immunoprecipitation-sequencing (ChIP-seq) experimental design. Cerebral cortex was dissected from embryonic day 18 (E18) rat brain and the hippocampus was removed. Primary cortical neurons were dissociated, seeded on day in vitro (DIV) 0 and injured on DIV8 with a p20 tip to generate injury gaps. Control and injured cortical neurons were collected on DIV9 and DIV10 respectively for ChIP-seq, followed by bioinformatic analysis. NGS, next generation sequencing. (**B**) Injured neuronal culture was fixed with 4% paraformaldehyde on DIV11 and subjected to immunofluorescence staining with anti-TUJ1 (neuron-specific class III beta-tubulin) antibody. Dotted white lines depict the borders of the injury gap. Re-growing neurites are shown extending into the gap. Scale bar, 100 µm.

Our laboratory previously characterized *WNT3A* as a promising RAG of which expression was induced during regeneration of injured cortical neurons [14]. Addition of recombinant WNT3A significantly promotes neurite re-growth of injured cortical neurons and organotypic brain slices. Intranasal administration of recombinant WNT3A to controlled cortical impact (CCI) TBI mice model increases the number of NeuN^+^ neurons and rehabilitates motor function based on behavior analysis. These findings strongly suggest WNT3A as a potential therapy for TBI. However, the mechanism underlying the injury-induced *WNT3A* expression is unclear. Since TBI has been reported to initiate transcriptomic and epigenomic reprograming in the brain [11,12], it is reasonable to anticipate that epigenetic regulation underlies the transcriptional induction of *WNT3A*. To this end, we analyzed ChIP-seq data of histone H3K4me3 and H3K27ac modifications between un-injured control and injured cortical neurons to investigate the mechanism of injury-induced *WNT*s expression. Primary cortical neurons were dissociated from hippocampi-removed cerebral cortices of embryonic day 18 (E18) rat and seeded onto poly-l-lysine-coated dishes on day in vitro (DIV) 0. To mimic mechanical injury to cortical neurons, a p20 tip was used to scrape lines over the neuronal culture, as the in vitro TBI model illustrated in Figure 1A. During regeneration, injured neurites re-grew into the injury gaps (Figure 1B). Based on our previous data, neuronal regeneration of injured cortical neurons are relatively active in 48 h upon injury [14]. Therefore, un-injured control or injured neuronal samples on DIV9 and 10 were collected respectively for ChIP-seq experiments.

**Figure 2:**
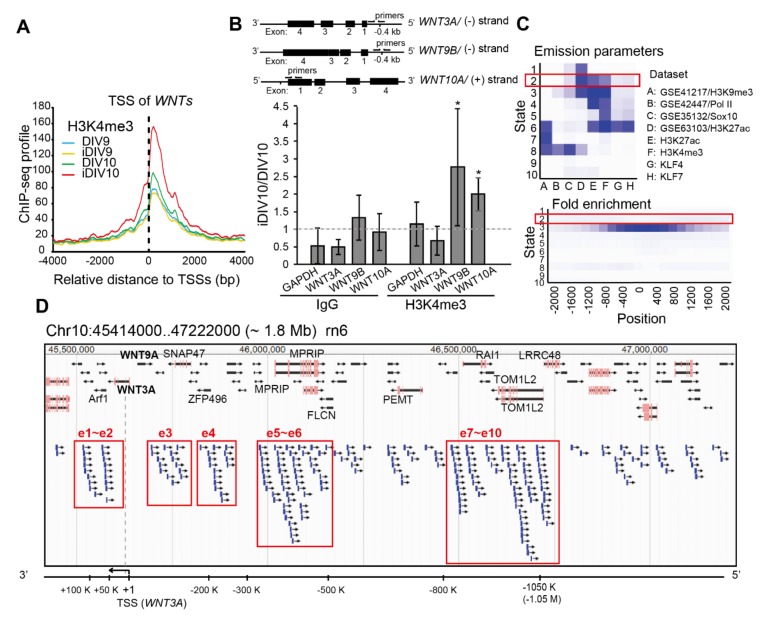
Prediction of putative enhancers of *WNT3A* gene. (**A**) Aggregation of normalized tri-methylation of histone H3 at lysine 4 (H3K4me3) signal density profiles of 88 WNT-related genes across the ±4 kb promoter regions. H3K4me3 signals across control and injured samples are indicated by colored lines. (**B**) Upper: Schematic diagrams of gene track for rat *WNT3A*, *WNT9B* and *WNT10A*. Targeted loci at the promoters for specific primers used for ChIP assays are shown. Bottom: The fold change of H3K4me3 at the promoters of *WNT3A*, *WNT9B* or *WNT10A* in cortical neurons comparing DIV10 and iDIV10 were analyzed by ChIP-qPCR. The fold change of H3K4me3 at *GAPDH* promoter was used as a control. Data were normalized to IgG and then to DIV10 controls, indicated as fold change. Data are presented as mean ± SEM from three independent experiments. Gray dotted line indicates the fold change = 1. Enrichment of IgG or H3K4me3 across control and injured samples at the promoter of each *WNT* were compared independently, using two-way ANOVA followed by Tukey’s test. *P ≤ 0.05. (**C**) Functional enrichment of chromatin states in rat genome performed by ChromHMM. Upper: Heatmap of the model parameter with chromatin states numbered in the emission order. The columns refer to relative enrichment for the indicated annotation in corresponding chromatin states. ChIP-seq data from four Gene Expression Omnibus (GEO) datasets as well as data from this study were used to train the model. Bottom: Heatmap of the positional enrichment of annotated chromatin states. The genomic feature of the State2 elements is indicated in red boxes. Enrichment of H3 lysine 27 acetylation (H3K27ac) (shown in blue), low RNA polymerase II (RNAPII) occupancy (shown in white) and deficient State2 elements residing within proximal promoter regions (shown in very light blue) suggest their enhancer identify. (**D**) Snapshot of JBrowse Genome Browser demonstrating the region across 1.8 Mb flanking the *WNT3A* transcription start site (TSS) of the rat genome (RCSC 6.0/*rn6*). Ten predicted genomic regions (e1-e10, indicated in boxed area) may be enhancers for the *WNT3A* gene based on the enrichment of clustered State2 elements assigned by ChromHMM. A diagram of *WNT3A* gene region is shown at the bottom.

The normalized H3K4me3 ChIP-seq profiles of WNT-related genes were aggregated across ±4 kb promoter regions with transcription start sites (TSSs) as the anchors (Figure 2A). The aggregated ChIP-seq signals aligned to the proximal promoters of WNT-related genes were increased up to 1.6-fold on injured DIV10 (iDIV10) compared to DIV10. ChIP-qPCR analyses for H3K4me3 at promoters of several *WNT*s among candidate RAGs were then performed. As shown in Figure 2B, active H3K4me3 mark at the promoter of *WNT9B* and *WNT10A* were increased, whereas that for *WNT3A* was not. If transcriptional regulation of *WNT3A* was not through promoter, it might be regulated by a distal enhancer.

**Figure 3:**
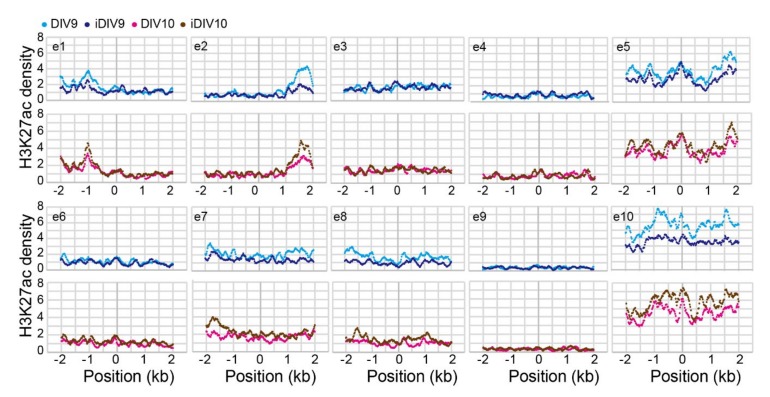
Aggregation of normalized H3K27ac ChIP-seq profiles for candidate *WNT3A* enhancer sub-regions. Normalized H3K27ac ChIP-seq profiles across the ±2 kb central base-pair within each candidate enhancer region (e1–e10) for control (DIV9, DIV10) and injured (iDIV9, iDIV10) samples are shown.

A number of computational tools have been employed to predict candidate enhancers during regeneration of injured cortical neurons. For example, ChromHMM, an approach for chromatin-state discovery and characterization [25], identified candidate enhancer regions based on extracting general chromatin features of enhancers. We classified the rat genome into 10 chromatin states, according to histone codes and the occupancy of transcription factors (Figure 2C). To do so, we utilized our reference datasets of H3K4me3, H3K27ac, Krüppel-like factor 4 (KLF4) and Krüppel-like factor 7 (KLF7) ChIP-seq profiles, as well as published ChIP-seq datasets of H3K9me3, RNA polymerase II (RNAPII), Sox10 and H3K27ac from the Gene Expression Omnibus (GEO) (GSE41217, GSE22878, GSE64703, GSE63103 and GSE64971) of multiple cell types in the nervous system for training chromatin-state models. Genomic elements with highly enriched H3K27ac marks (active enhancers) and low occupancy of RNAPII (Figure 2C, upper panel) that are distal to the TSS (Figure 2C, bottom panel), annotated as State2, are likely to feature enhancer regions. We then defined ten genomic regions within a 1.8-Mb interval flanking the *WNT3A* TSS in the rat genome (RCSC 6.0/*rn6*). These regions, e1 to e10, represented putative enhancer domains that may regulate the expression of *WNT3A* gene. Each predicted domain contains a high density of State2 genomic elements assigned by ChromHMM (Figure 2D). Notably, this 1.8-Mb region also includes the *WNT9A* gene, and it is possible that *WNT3A* and *WNT9A* share common enhancers for coordinated transcriptional control. To screen for enhancer regions that are active during neuronal regeneration, H3K27ac ChIP-seq signals were evaluated. As shown in Figure 3, H3K27ac signals mapped to each putative enhancer domain were aggregated. The ChIP-seq signal density for H3K27ac was elevated at the e2, e5, e7, e10 enhancer regions in iDIV10 samples compared to DIV10. The H3K27ac modification in e1 and e8 were slightly increased, whereas no obvious change could be observed in e3, e4, e6 and e9 regions. In contrast, H3K27ac modification in e1-e10 was generally decreased or remained unchanged throughout the enhancer regions on iDIV9 compared to DIV9. Thus, the candidate enhancer for induced *WNT3A* expression may lie within e2, 5, 7 and 10.

**Figure 4:**
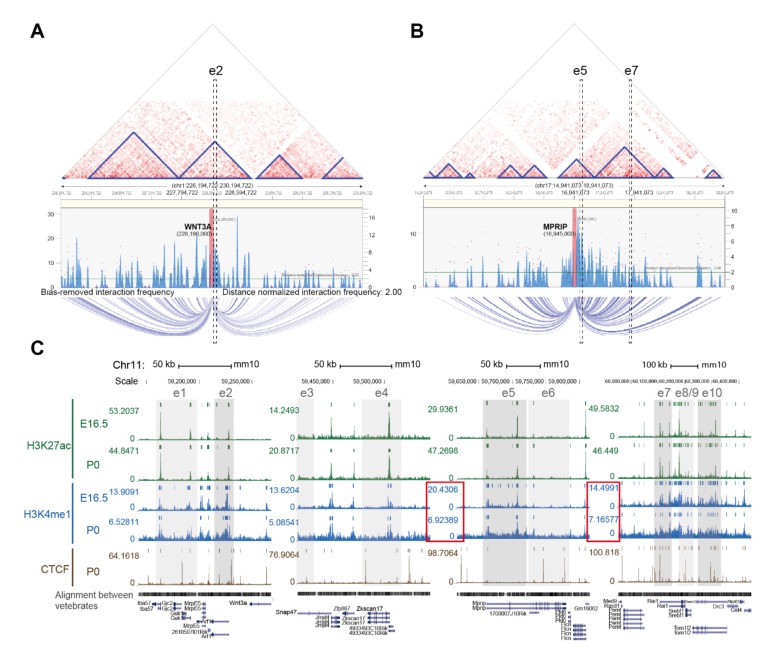
Interactions of transcriptional activation domains and histone modifications at the e5 and e7 enhancers. (**A**, **B**) Visualization of identified chromatin interactions around (**A**) e2/*WNT3A* and (**B**) e5/e7 regions. Blue triangles in the interaction heatmaps indicate topologically associating domains (TADs) that form 3D structural compartments of the genome. Interaction frequency graphs, consisting of the blue bars and magenta dots, represent bias-removed and distance-normalized interaction frequencies, respectively. Arc-diagrams anchored by indicated loci (*WNT3A* and *MPRIP* genes, indicated by magenta bars in the frequency graphs) are defined by indicated threshold (distance-normalized interaction frequencies ≥ 2) and shown. (**C**) Homologous loci of predicted enhancer domains aligned with the mouse genome assembly. Chromosomal positions for known histone H3K4me1 and H3K27ac modifications, and CCCTC-binding factor (CTCF) binding profile during mouse forebrain development (E16.5-P0) are shown. ChIP-seq data of the indicated samples were collected from the ENCODE database and visualized in UCSC Genome Browser. Colored bars above peaks in each track indicate confidence of enrichment for the corresponding genomic feature. Putative enhancers are shown in gray shadowed areas. e2, e5, e7, e10 are highlighted by darker gray shadows. Relative H3K4me1 signals at e5-e6 and e7-e10 during mouse forebrain development (E16.5-P0) are indicated (red boxed region). Genomic sequence alignment among vertebrates and gene tracks are depicted below ChIP-seq profiles.

While the predicted enhancers for the *WNT3A* gene reside within the region from +100 kb to -1 Mb to the TSS (e1-e10), it is possible that the regulation of *WNT3A* expression is governed by spatial chromatin interaction between functional genomic elements, for instance, promoter-enhancer interaction. To investigate possible interactions between *WNT3A* promoter and the candidate enhancers, putative interaction was shown based on the 3D Interaction Viewer and database (3DIV) online tool (https://www.kobic.kr/3div/) that collects publicly available Hi-C data of human cells and provides normalized chromatin frequencies to interpret genome-wide chromatin interactions [26]. As shown in Figure 4A, several topologically associating domains (TADs) were identified within an interaction range of 2 Mb around *WNT3A* in the genome of human hippocampal tissue [27]. TADs are structural building blocks of DNA that have been proposed to form through loop extrusion of interphase chromosome, stabilized by the binding of boundary elements and loop-extruding factors (e.g., CTCF, cohesin) [28]. Insulated spatial DNA compartments restrict the interplay between functional regulatory domains within the same TAD and allow specific promoter-enhancer pairs for sufficient gene activation [29]. The genomic region homologous to the e2 enhancer in the rat genome resides within one of the TADs that comprises *WNT3A* gene and presents relatively high interaction frequency to *WNT3A*. Moreover, according to the GeneHancer database, an integrated human enhancer database that infers enhancer-gene associations [30], also suggests a high likelihood score of this region as potential elite promoter/enhancer for *WNT3A* gene. The relative position of e2 to *WNT3A* is similar between human and rat, with the TSS distance of +79.5 kb and +57.8 kb to the e2 region, respectively. Through mapping the homologous loci of rat e5-e7 region to the human genome, a serendipitous finding revealed an evolutionary shift of e5-e7 region to a different chromosome in human (chromosome 17), different from e2 region and *WNT3A* gene (chromosome 1). Nonetheless, the homologous e5 and e7 regions reside within two separated TADs encompassed in a larger building block and the data suggests an interaction between e5 and e7 (Figure 4B). This finding uncovers a possibility of long-range inter-chromosomal regulation of *WNT3A* gene in human.

While there is currently no publicly accessible ChIP-seq database for rat brain, we compared ChIP-seq datasets for H3K27ac and H3K4me1 enhancer marks in mouse forebrain that are available in the ENCODE database (https://www.encodeproject.org/) [31]. Annotations of these enhancer domains homologous between rat and mouse genome were shown in Figure 4C [32,33]. There is a clear decrease of the H3K4me1 mark in these regions from E16.5 to postnatal day 0 (P0), according to ChIP-seq data. Normalized signal of H3K4me1 mark in the e5-e6 regions reduced to 1/3 (20.4 to 6.9) during development and to 1/2 (14.5 to 7.1) at e7 regions (Figure 4C, red boxes); however, the level of H3K27ac mark remained relatively constant over the same time period. This differential change of histone modification suggests that the H3K4me1 may be the decisive mark. In other words, H3K4me1 may prime and activate these enhancers during development and could be a cue for RAG regulation during neuronal regeneration. The existence of CCCTC-binding factor (CTCF) sites surrounding e1-e2, e4, e5-e6 and e7-e10 regions probably reflects a functional role of the CTCF architectural protein in bridgingenhancer-promoter interacting topology(Figure 4C) [34,35].

### 2.2. Enhancer-Mediated WNT3A Expression through Histone Modification and Chromosome Topological Transformation

**Figure 5:**
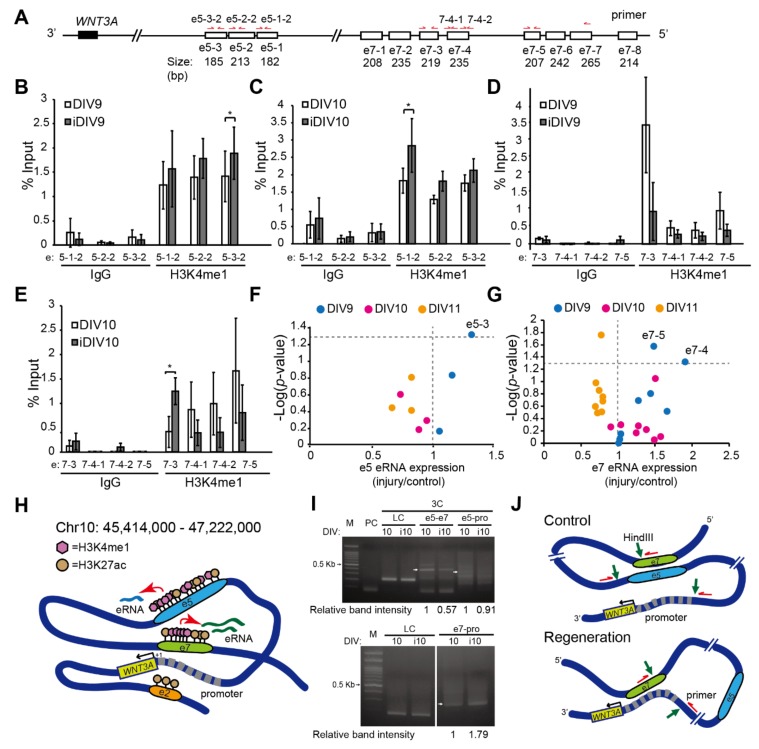
H3K4me1 modifications, enhancer RNAs (eRNAs) and topological transformation at *WNT3A* enhancer/promoter regions during neuronal regeneration. **(A)** Schematic diagram of primers design for ChIP-qPCR assays at e5 and e7 regions. (**B–E**) ChIP-qPCR was performed to examine the relative H3K4me1 modifications at the e5 (**B,C**) and e7 (**D,E**) regions. IgG enrichment was used as a negative control. Samples were from cortical neurons on DIV9, iDIV9, DIV10 and iDIV10, respectively. Data are mean ± SEM (*n* = 3 and *n* = 4 for experiments on DIV9 and DIV10, respectively). *P ≤ 0.05 (two-way ANOVA followed by Tukey’s test, comparing the enrichment of IgG or H3K4me1 between control and injured samples at the indicated time points). (**F,G**) Volcano plots displaying differential expression (DE) of e5 and e7 eRNAs between control and injured samples on DIV9, DIV10 and DIV11. The x-axis represents the fold change of eRNA expression, which was calculated based on the results of RT-PCR. The y-axis corresponds to –log (*P*-value), determined by paired Student’s *t*-test, comparing e5 or e7 eRNA expressions between control and injured sample at the indicated time points. Vertical gray dotted line represents the value of DE = 1, while the horizontal gray dotted line borders the cut-off value of –log (*P* = 0.05). eRNA DE values are shown as mean from at least three independent experiments (e5: *n* = 3; e7: *n* = 4). (**H**) Schematic summary of histone H3K4me1- and eRNA-mediated enhancer regulation for *WNT3A* transcription during regeneration. Dark blue line depicts genomic DNA ranging from predicted enhancers e1 to e10. Relative position of the e2, e5, e7 enhancer regions are denoted. Pink hexagon represents H3K4me1. Brown circle represents H3K27ac. eRNAs derived from e5 and e7 are indicated as light blue and green lines, respectively. Red arrows point to the activation of eRNA transcription mediated by increased histone modification during regeneration. Black arrow marks the transcription of *WNT3A*. Promoter region for *WNT3A* is depicted by thick gray dashed line. The transcription start site (TSS) is denoted as “+1”. (**I**) Chromosome conformation capture (3C) assays determine the topological interaction among e5, e7 enhancers and the promoter of *WNT3A*. PCR products amplified using the indicated primers after 3C assays were shown in DNA gel electrophoresis. If interaction exists, the expected DNA size would be e5-e7 (347 bp), e5-promoter (pro) (336 bp) and e7-pro (271 bp). M: marker. PC: primer control. LC: loading control. Relative intensity of e5-e7, e5-pro or e7-pro interaction was quantified based on the band intensity (Gel-Pro Analyzer 3.1). Values are first normalized to LCs then to DIV10 un-injured samples and denoted at the bottom of each DNA gel image. (**J**) Schematic model of topological transformation of enhancer-promoter looping mechanism for *WNT3A* regulation during neuronal regeneration. Green arrows point to HindIII cutting sites. Red arrows indicate the site and direction of primers designed for 3C.

To examine the role of the H3K4me1 modification at the putative enhancers of *WNT3A*, un-injured or injured cortical neurons were collected to perform ChIP-qPCR assays of H3K4me1 modification at the e5, e7, e10, respectively. The H3K4me1 modification at e5 sub-regions, e5-1 and e5-3, was significantly increased on iDIV9 or iDIV10 compared to un-injured controls (Figure 5A-C). Similarly, the H3K4me1 mark at e7 sub-region, e7-3, was significantly increased during regeneration on iDIV10 (Figure 5A,D,E). H3K4me1 modification at e10, on the other hand, was not detectable via qPCR. Recent studies linked the expression of non-coding enhancer RNAs (eRNAs) to functional enhancers [36,37,38], thus the expression of cognate eRNAs derived from the e5 and e7 were also examined. As shown in Figure 5F,G, the relative expression of e5-3, e7-4, e7-5 eRNA was significantly increased on iDIV9. To exclude the possibility that these eRNAs are transcribed from the transcripts of proximal coding genes, we performed qPCR for expression of *MPRIP*, which is located within the e5 region, and concluded no change of the gene during regeneration (Figure S1). Together, these results suggest that the H3K4me1-modulated e5 (chr10: 46,023,200–46,097,800) and e7 (chr10: 46,518,000–46,553,800) regions, containing TADs and exhibiting increased eRNAs, may be active enhancers for *WNT3A* expression during regeneration. We propose that these eRNAs may recruit transcriptional components allowing e5, e7 enhancer-promoter looping to coordinate *WNT3A* gene expression (Figure 5H). These eRNAs may stabilize the chromosome looping between enhancer sub-regions and enhancer-promoter to control *WNT3A* transcription [39].

To test the possible interaction between e5 and e7 regions (Figure 4B), we then performed chromosome conformation capture (3C) assays to examine topological interaction between e5 and e7 (e5-e7), e5 and *WNT3A* promoter (e5-pro), e7 and *WNT3A* promoter (e7-pro) during regeneration of injured cortical neurons. Primary cortical neurons were fixed and collected on DIV10 and iDIV10 respectively for HindIII restriction enzyme digestion. Following DNA ligation, interacting genomic regions were then amplified via PCR and resolved by DNA gel electrophoresis. As shown in Figure 5I, there were interactions between e5-e7, e5-pro and e7-pro before injury, as the indicative bands were clearly seen in the DNA gel. During regeneration, the interaction between e5-e7 and e5-pro decreased, whereas e7-pro interaction increased. These findings suggest that e5 and e7 regions, with low histone H3K4me1 modifications, are brought to the proximity of *WNT3A* promoter via DNA looping in un-injured cortical neurons. Under this topological conformation, low *WNT3A* is expressed (Figure 5J, upper panel). Neuronal injury triggers change of epigenome and topological transformation of chromosome to coordinate expression of candidate RAGs. During regeneration, histone H3K4me1 modifications are increased at e5 and e7, leading to reduced e5-e7 interaction (Figure 5B-E; Figure 5I, upper panel). This topological transformation facilitates the looping between e7 and *WNT3A* promoter, likely through the help of induced eRNAs, and leads to induced *WNT3A* expression (Figure 5F,G; Figure 5J, bottom panel).

## 3. Discussion

**Figure 6:**
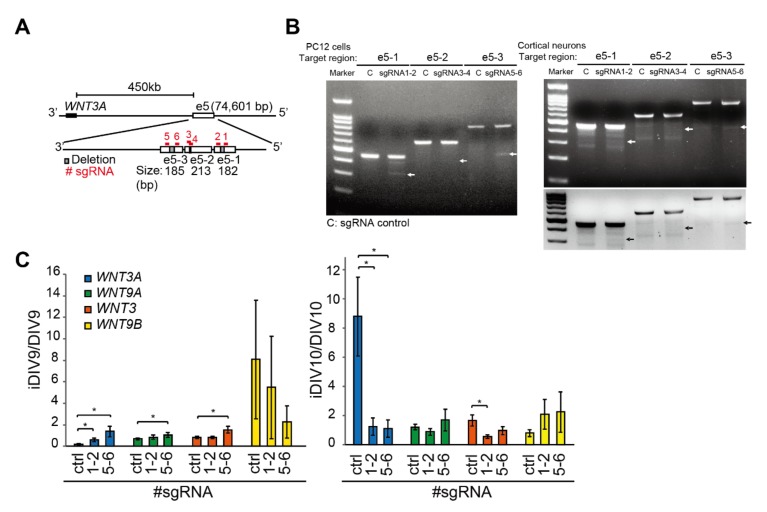
Genome editing to disrupt the e5 enhancer region repressed injury-induced expression of *WNT3A*. (**A**) Schematic diagram of the single guide RNAs (sgRNAs) design for CRISPR (clustered regularly interspaced short palindromic repeats)/Cas9-mediated e5 deletion. Six sgRNAs specifically targeting the sub-regions of e5 (e5-1, e5-2, e5-3) are represented as red bars in the enlarged view of e5. Gray bars represent the enhancer deletion regions targeted by each pair of sgRNAs. (**B**) CRISPR/Cas9-mediated enhancer deletions were detected by T7 endonucleaes I (T7EI) assays in PC12 cells and cortical neurons. Left: PC12 cells were transfected with pAll-Cas9.pPuro constructs targeting the e5 sub-regions to generate insertion/deletions (indels). Right: Primary cortical neurons were infected with pAll-Cas9.pPuro lenti-constructs at MOI = 0.25 on DIV7. Genomic DNA was isolated on DIV10 and subjected to PCR amplification, product re-annealing, T7EI digestion and DNA electrophoresis. White and black arrows point to anticipated T7EI-digested DNA fragments, indicating successful enhancer editing. The bottom panel demonstrates color-inversed image for better visualization. (**C**) *WNT3A* (DIV9: *n* = 5; DIV10: *n* = 4), *WNT9A* (*n* = 4), *WNT3* (*n* = 4), *WNT9B* (DIV9: *n* = 4; DIV10: *n* = 3) expressions were assessed by qPCR after CRISPR/Cas9 editing at the e5 sub-regions. The values were calculated as the ratio of iDIV9/DIV9 (left panel) or iDIV10/DIV10 (right panel). Data are presented as mean ± SEM. *P ≤ 0.05 (paired Student’s *t*-test, comparing the fold change of individual *WNT* expressions during regeneration between control and e5-edited samples).

Since H3K4me1 modification was previously shown to be enriched at active enhancers and to fine-tune transcriptional activity by recruiting chromatin modifiers [40], we reasoned that the increase of H3K27ac at *WNT3A* enhancer on iDIV10 (ChIP-seq results) could be a consequence of H3K4me1-primed enhancer activation (Figure 3). In support of this possibility, histone ChIP-qPCR assays demonstrated that H3K4me1 at the e5 region was increased as early as iDIV9, concomitant with increased eRNA transcripts (Figure 5B,C and F). This study has thus characterized a preceding role of H3K4me1 that predominate the transcriptional activity of e5/e7 under the context of neuronal regeneration (Figure 3). If e5/e7 is a critical enhancer region for *WNT3A* gene, one would expect that injury-induced *WNT3A* expression would be affected by disruption of these sequences. To test this possibility, six single guide RNAs (sgRNAs) were designed to target the e5 region, and CRISPR (clustered regularly interspaced short palindromic repeats)/Cas9-mediated genome editing using pairs of sgRNAs was performed to generate 50-bp, 4-bp and 83-bp deletions in the enhancer (Figure 6A; see Supplementary Methods). T7 endonuclease I (T7EI) cleavage assays were used to assess the editing efficiency. As shown in Figure 6B, sgRNA1-2 and sgRNA5-6 successfully generated deletions in PC12 cells and primary cortical neurons, whereas sgRNA3-4 appeared to be less efficient. After treatment of cortical neurons with sgRNA1-2 and sgRNA5-6 to disrupt e5-1 or e5-3, the expressions of *WNT3*/*WNT9A* gene cluster and its paralog, *WNT3*/*WNT9B* cluster, were examined (see Supplementary Methods). Specifically, injury-induced *WNT3A* and the paralog *WNT3* expression on iDIV10 were significantly reduced on iDIV10 (Figure 6C, right panel) while slightly increased expression of *WNT3A*, *WNT9A* and *WNT3* was observed on iDIV9 (Figure 6C, left panel). These data suggest that the e5 is likely involved in transcriptional regulation of *WNT3A* expression.

While H3K4me1 modification primes/activates enhancer collaboratively with other histone modifications (such as H3K27ac) and plays a decisive role in enhancer activation, the cellular mechanism underlies this transcriptional regulation is under-studied [41]. The enzymatic activity of specific H3K4 methyltransferases, such as the members of the MLL/COMPASS family, MLL3/4, are responsible for H3K4me1 on enhancers during cell differentiation and the pathogenesis of cancer [42,43,44]. Augmentation of H3K4me1 on enhancers serves as the binding hub for the recruitment of transcription factors and other effector proteins that remodel chromatin environment and the assembly of transcriptional machinery [45]. Recent studies demonstrated that catalytically deficient MLL3/4 and knockout of MLL3/4 in cell lines lead to reduction of H3K27ac and target gene expression respectively [46] and that reduced binding of chromatin remodeling complex, BRG1/BRM-associated factor (BAF), to enhancer is associated with the depletion of H3K4me1 in mutant mouse embryonic stem cells (ESCs) with catalytically inactive MLL3/4 [47]. It is likely that e5/e7 enhancer accommodates methyltransferases MLL3/4 as well as candidate effector proteins to transcriptionally activate *WNT3A* gene during regeneration of injured cortical neurons. In this regards, further characterization of the enzymatic activity/expression level of MLL3/4 and potential transcriptional factor binding at the e5/e7 region during neuronal regeneration would reveal additional regulation, providing therapeutic perspectives for developing TBI treatment in clinic.

## 4. Materials and Methods

### 4.1. Animals and Ethics Approval

All experiments were executed in accordance with the guidelines of the Laboratory Animal Center of National Tsing Hua University (NTHU; No. 101, Section 2, Kuang-Fu Road, Hsinchu, Taiwan), and protocols were approved by the NTHU Institutional Animal Care and Use Committee (approval# 10658, approved on 01 April 2018).

### 4.2. Reagents

Powder of Minimum Essential Medium (MEM), fetal bovine serum (FBS), horse serum (HS), penicillin-streptomycin (PS), B-27™ supplement, L-glutamine (L-Gln), Antibiotic-Antimycotic (AA and TRIzol reagent were purchased from Invitrogen (Carlsbad, CA, USA). Neurobasal^®^ medium was purchased from Gibco (Grand Island, NY, USA). Poly-L-lysine (PLL), glutamate and Cytosine-β-D-arabinofuranoside (AraC) were from Sigma-Aldrich (Saint Louis, MO, USA). Anti-H3K4me3 antibody (39159) was purchased from Active Motif (Carlsbad, CA, USA). Anti-H3K27ac (ab4729) and anti-H3K4me1 (ab8895) antibodies were from Abcam (Cambridge, CB2 0AX, UK). T4 Ligase was purchased from New England BioLabs (Ipswich, MA, USA).

### 4.3. In Vitro Culture of Primary Neurons and the TBI Model

Sprague Dawley (SD) rats were purchased from BioLASCO Taiwan Co., Ltd. Primary cortical neurons were dissociated from dissected cortices of rat embryos (E18), seeded onto PLL-coated dishes and culture in vitro as previously described [48,49]. For the in vitro TBI model, cortical neurons were injured by scraping with a p20 tip to generate gaps over the culture on DIV8 (Figure 1A). During neuronal regeneration, re-growing neurites extend into the injured gaps as previously demonstrated [14], and the neuronal cultures were then subjected to further experimental approaches.

### 4.4. ChIP-Seq

For ChIP-seq, ChIP was performed as previously described [50]. Briefly, primary cortical neurons were injured on DIV8 by scraping lines on the culture with a p20 tip to mimic mechanical injury. Un-injured control or injured neurons were fixed on DIV9 and DIV10 respectively with 1% (v/v) formaldehyde; glycine was added to quench the cross-linking reaction. Lysates were sonicated to produce 200-500-bp DNA fragments. Sheared chromatin was pulled down by anti-H3K4me3 or H3K27ac antibodies. DNA was purified and DNA libraries were prepared using the TruSeq^TM^ ChIP Library Prep Kit (Illumina, San Diego, CA, USA) and sequenced on an Illumina HiSeq 2500 System as 2 × 100-bp paired ends. Sequencing was performed at the Next Generation Sequencing (NGS) High Throughput Genomics Core (Academia Sinica, Taiwan). Adaptor trimming was performed using PEAT (https://github.com/jhhung/PEAT) [51], followed by reads alignment to the rat genome (*Rnor 6.0*/*rn6*) using Bowtie v1.1.1 (https://sourceforge.net/projects/bowtie-bio/files/bowtie/1.1.1/) [52] with the following parameters (-n 1 --best -l 100 -strata -a -M 1). Peak calling was performed by MACS v1.4.2 (https://github.com/taoliu/MACS/) to identify histone ChIP-seq enrichment over input with the following parameters (nomodel, shiftsize 73, *P*-value = 0.01) [53].

### 4.5. Data Analysis

#### 4.5.1. Aggregation Plot

To visualize the overall enrichment and signal distribution of H3K4me3 across gene promoters in each sample, we aggregated normalized ChIP-seq signals across a ±4 kb region flanking the TSSs of 88 WNT-related genes. Normalized ChIP-seq signals were calculated as reads per million (RPM).

#### 4.5.2. ChromHMM for Enhancer Prediction

To predict enhancers, ChromHMM was used for chromatin-state discovery on the basis of chromatin modification patterns by model training and analysis [25]. ChIP-seq datasets utilized for learning chromatin-state models were collected from our H3K4me3, H3K27ac, KLF4 and KLF7 ChIP-seq profiles of control and injured cortical neurons (on DIV9 and DIV10), and GEO accession numbers: GSE41217 (H3K9me3 modification in hippocampal neurons), GSE22878 (RNAPII occupancy in cortical neurons), GSE64703 (Sox10 occupancy in spinal cord), GSE63103 (H3K27ac modification in Schwann cells) and GSE64971 (H3K27ac modification in injured sciatic nerves). Due to limited numbers of publicly available rat ChIP-seq datasets, data of multiple cell types and tissues were included as training datasets for enhancer prediction to improve performance. Processed BED files containing genomic information were used as the input for chromatin-state annotation. Genome-wide chromatin was segmented into 10 chromatin states. To identify potential enhancers, modeled emission parameter and positional enrichment profiles were referenced. High enrichment of H3K27ac and relatively low H3K4me3 and RNAPII at distal regions to the TSSs indicated chromatin regions that were annotated as State2, and these regions were selected as putative enhancers. Positional information of the segments in BED files were uploaded onto JBrowse Genome Browser for visualization.

#### 4.5.3. Long-Range Chromatin Interaction Prediction

To predict possible interaction between putative enhancers, publicly available Hi-C datasets of human hippocampal tissues (sample GSM2322543 from series GSE87112) [27] were analyzed in the 3D-genome Interaction Viewer and database (3DIV; https://www.kobic.kr/3div/) [26]. The “Interaction visualization” function was used with query gene names of *WNT3A* and *MPRIP*, the gene located within the e5 enhancer region, to visualize chromatin interactions around *WNT3A* gene and predicted enhancer e5-e7 region, respectively.

#### 4.5.4. ENCODE Datasets Processing

ChIP-seq datasets were collected from the ENCODE Project Consortium (https://www.encodeproject.org/) [54] and visualized on UCSC Genome Browser (https://genome.ucsc.edu/) with the alignment to mouse genome assembly (GRCm38/mm10). H3K27ac and H3K4me1 ChIP-seq data of mouse forebrain tissue were extracted from ENCSR428OEK (H3K27ac; E16.5), ENCSR094TTT (H3K27ac; P0), ENCSR141ZQF (H3K4me1; E16.5), ENCSR465PLB (H3K4me1; P0). CTCF ChIP-seq data of mouse forebrain tissue was extracted from ENCSR677HXC (CTCF; P0). Multiz alignments and conservation track are shown. Homologous loci of putative enhancers e1-e10 between rat and mouse genome were aligned and indicated in the shadowed regions in the track view (shown in Figure 4C).

### 4.6. ChIP-qPCR and eRNA Quantification

For histone ChIPs, DIV8-injured or un-injured control cells were cross-linked on DIV9 and DIV10 with 0.8% formaldehyde. Chromatin was sonicated to generate 200-500-bp fragments for subsequent immunoprecipitation of histone-DNA complexes using anti-H3K4me3, anti-H3K4me1, or anti-rabbit IgG antibody-conjugated protein A. DNA was purified in the presence of RNase A and Proteinase K, followed by phenol/chloroform DNA extraction. The immunoprecipitated DNA and input control were analyzed by qPCR, with specific primers for *WNT3A*, *WNT9B* and *WNT10A* promoter regions and putative enhancers e5, e7 and e10 for *WNT3A*. The enrichment of histone modification at promoters or enhancers was determined as percentage of input (% input) = 100 × 2^{Ct(ChIP) – [Ct(input) - log2(input dilution factor)]}.

For eRNA quantification, total RNAs were isolated from DIV8-injured or un-injured cortical neurons from DIV8-11 for reverse transcription polymerase chain reaction (RT-PCR). eRNAs of interest were amplified by specific primers and analyzed by DNA gel electrophoresis. Band intensities were quantified by Gel-Pro Analyzer 3.1 and normalized to *GAPDH*. (https://gel-pro-analyzer.software.informer.com/3.0/)

### 4.7. Chromosome Conformation Capture (3C) Analysis

For 3C template, primary cortical neurons were fixed with 0.8% formaldehyde. Glycine was added to quench the reaction. Cells were centrifuged and the pellet was lysed [10 mM Tris–HCl pH 8.0, 10 mM NaCl, 0.2% Igepal CA-630 (NP40), with protease inhibitors], incubated on ice for 15 min, followed by centrifugation. NEB 2.1 buffer containing 0.3% of SDS and subsequently 1.6% of Triton X-100 were added to the pellet and incubated at 37 ºC on a shaker. Samples were digested with HindIII overnight at 37 °C. 1.4% of SDS quenched enzymatic digestion at 65 °C for 30 min on a shaker. Ligation using T4 ligase was performed at 4ºC overnight followed by proteinase K and RNase A treatment. DNA was purification and subjected to PCR with specific primers and analyzed by gel electrophoresis.

Primers used for 3C:

Promoter: 5′-GGGGCCAGGGTCTATAAAAACATGGATG3-3′;

e5: 5′-CTGTTGGCACCTTCTTTCTGCTCTCTGG-3′;

e7: 5′-GAAAGAGATGAGGGCACAGTGAAGGAGG-3′;

LC1 forward: 5′-ACAAGCGAGAGCTAGGACACC-3′;

LC1 reverse: 5′-ACTCTTACAAGTTGGCCTTCACTT-3′;

LC2 forward: 5′-CCATGGCTTTGATCTTCAAGAACAG-3′;

LC2 reverse: 5′-CCAAGGGATAAGTAGCCTGTGTG-3′

### 4.8. Statistical Analysis

All results are expressed as mean ± SEM from at least three independent experiments unless otherwise noted. Paired Student’s *t*-test was performed using Microsoft^®^ Excel^®^ 2016 MSO (16.0.4849.1000) 64 bits. Prism (v.8.0.2; GraphPad Software; https://www.graphpad.com/scientific-software/prism/) was used to perform two-way ANOVA followed by Tukey’s test. Statistical significance (*) is defined as *P* ≤ 0.05.

## 5. Conclusions

Together, we have identified a novel enhancer for *WNT3A* gene expression. In response to neuronal injury, H3K4me1-mediated enhancer activation and induced eRNAs transform DNA topology via looping sub-regions of this enhancer to the proximity of promoter to drive *WNT3A* gene expression during regeneration. We conclude that this novel enhancer−promoter looping mechanism is responsible for the induced *WNT3A* gene transcription during regeneration of injured cortical neurons.

## Supplementary Materials

The following are available online at https://www.mdpi.com/1422-0067/21/5/1891/s1.

## Abbreviations

TBI	Traumatic brain injury
RAG	Regeneration-associated gene
H3K27ac	Histone 3 lysine 27 acetylation
H3K4me1/3	Histone3 lysine 4 mono-/tri-methylation
ChIP-seq	Chromatin immunoprecipitation-sequencing
3C	Chromosome conformation capture
CTCF	CCCTC-binding factor
TAD	Topologically associating domain
eRNA sgRNA	Enhancer RNA Single guide RNA
