# Supplementary material for: Epigenetic Regulation of WNT3A Enhancer during Regeneration of Injured Cortical Neurons
Source: Int J Mol Sci. 2020 Mar 10;21(5):1891. doi: 10.3390/ijms21051891 (PMC7084788; doi:10.3390/ijms21051891)
Supplement: Supplementary file 1 [file ijms-21-01891-s001.pdf]

Supplementary Methods and Figures

1. Supplementary Methods

CRISPR/Cas9 enhancer editing

Six sgRNAs targeting the putative enhancer e5 used in this study were designed and cloned into All-in-One system sgRNA/Cas9 expression lentivector (pAll-Cas9.pPuro) individually by RNAi Core Facility (Academia Sinica). Human codon-modified *Cas9* gene and the sgRNAs were driven by CMV and human U6 promoter, respectively. sgRNA1 and sgRNA2 target e5-1 region and were further cloned into a single construct; sgRNA3 and sgRNA4 target e5-2 region and were further cloned into a single construct; sgRNA5 and sgRNA6 target e5-3 region.

For CRISPR/Cas9 enhancer editing, primary cortical neurons were infected with lentivirus expressing CRISPR constructs (control /or sgRNA1-2 /or sgRNA3-4 /or sgRNA5 and 6) at MOI = 0.25 on DIV7 for overnight. Genomic DNA of infected neurons were purified on DIV10 for T7 endonuclease I (T7EI) cleavage assay. Total RNA from infected neurons were extracted on DIV9 or DIV10 for gene expression analysis. For CRISPR/Cas9-editing in PC12 cells, PC12 cells were obtained from the ATCC and cultured on collagen-coated dishes in Dulbecco's Modified Eagle Medium (DMEM, Carlsbad, CA, USA) medium supplemented with 1% L-Gln, 5% FBS and 10% HS in 10% CO_2 condition. PC12 cells were transfected with CRISPR constructs by using Lipofectamine 2000 (Invitrogen) for 5 h according to the manufacturer's instructions. Puromycin (5 μg/ml) was added for selection. Genomic DNA was extracted after five days for the subsequent T7EI cleavage assays.

To examine the result of CRISPR/Cas9 editing, genomic DNAs were purified from PC12 cells or cortical neurons following the manufacturer's protocol of Wizard® Genomic DNA Purification Kit (Cat# A1120; Promega, Madison, WI, USA). Extension of targeted deleted loci were amplified from purified genomic DNA via PCR by using specific primer pairs. Purified PCR products were subjected to perform template annealing and T7EI digestion, following the manufacturer's instruction (Cat# M0302; New England BioLabs, Ipswich, MA, USA). The results of T7EI assays were resolved by DNA gel electrophoresis.

Primers used for T7EI assay

Edited enhancer region	Forward primer (5'-3')	Reverse primer (5'-3')
e5-1 CRISPR	CCATGGCTTTGATCTTCAAGAACAG	ATGAGAGGGAGATCTTGTTTCTTCC
e5-2 CRISPR	AATATCTGTGAGCTTCTGCTGGC	CTCACCCACTTGTCTGTCTCAC
e5-3 CRISPR	AATATCTGTGAGCTTCTGCTGGC	TCCCATATGAAGGGCTGTGACTT

2. Supplementary Figure

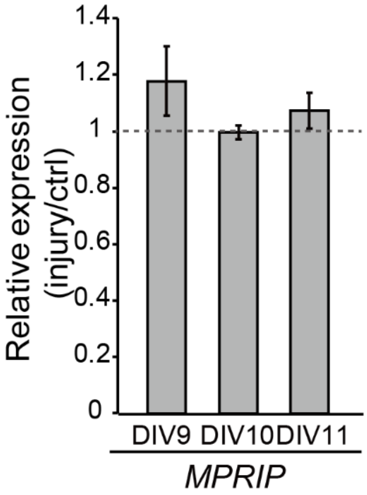

Figure S1. Analysis of differential expression of the e5-overlappingt gene, *MPRIP*, by qPCR during neuronal regeneration. Un-injured control or DIV8-injured primary cortical neurons were collected on DIV9, 10, 11, respectively, and subjected to RT-qPCR analysis. Data are presented as mean ± SEM from three independent experiments. (data was analyzed using paired Student's *t*-test to compare the gene expression between control and injured samples at the indicated time points)
